# Multicenter Validation of the English Version of the Dépistage Cognitif de Québec: a Cognitive Screening Tool for Atypical Dementias

**DOI:** 10.1093/arclin/acae092

**Published:** 2024-10-11

**Authors:** Synthia Meilleur-Durand, Marianne Lévesque, Frederic St-Onge, Mario Masellis, Ging-Yuek Robin Hsiung, Pamela Jarrett, Sylvia Villeneuve, Gabriel Léger, David Salmon, Doug Galasko, Stephen C Cunnane, Serge Gauthier, Brandy Callahan, Leila Sellami, Carol Hudon, Joël Macoir, Louis Verret, Alison Cassivi-Joncas, Michael Comishen, Robert Laforce

**Affiliations:** Département des Sciences Neurologiques, Clinique Interdisciplinaire de Mémoire (CIME) du CHU de Québec, Quebec, Canada; Département des Sciences Neurologiques, Clinique Interdisciplinaire de Mémoire (CIME) du CHU de Québec, Quebec, Canada; McGill University, Department of Psychiatry, Faculty of Medicine, Montreal, Canada; Sunnybrook Health Sciences Centre, Department of Neurology, Toronto, Canada; University of British Columbia, Department of Neurology, Vancouver, Canada; Horizon Health Network, Department of Geriatrics, Dalhousie University, Halifax, Canada; McGill University, Department of Psychiatry, Faculty of Medicine, Montreal, Canada; University of California San Diego, Department of Neurology, La Jolla, USA; University of California San Diego, Department of Neurology, La Jolla, USA; University of California San Diego, Department of Neurology, La Jolla, USA; Research Center on Aging, Department of Geriatrics, Sherbrooke University, Sherbrooke, Canada; McGill University, Department of Psychiatry, Faculty of Medicine, Montreal, Canada; Hotchkiss Brain Institute, Department of Psychology, University of Calgary, Calgary, Canada; Département des Sciences Neurologiques, Clinique Interdisciplinaire de Mémoire (CIME) du CHU de Québec, Quebec, Canada; Centre de Recherche CERVO, Department of Psychology, Université Laval, Quebec, Canada; Centre de Recherche CERVO, Department of Psychology, Université Laval, Quebec, Canada; Département des Sciences Neurologiques, Clinique Interdisciplinaire de Mémoire (CIME) du CHU de Québec, Quebec, Canada; Département des Sciences Neurologiques, Clinique Interdisciplinaire de Mémoire (CIME) du CHU de Québec, Quebec, Canada; Sunnybrook Health Sciences Centre, Department of Neurology, Toronto, Canada; Département des Sciences Neurologiques, Clinique Interdisciplinaire de Mémoire (CIME) du CHU de Québec, Quebec, Canada

**Keywords:** Test construction, Assessment, Alzheimer’s disease, Frontotemporal dementia, Norms/normative studies, Cognitive screening

## Abstract

**Background:**

Early detection of atypical dementia remains difficult partly because of the absence of specific cognitive screening tools. This creates undue delays in diagnosis and management. The Dépistage Cognitif de Québec (DCQ; dcqtest.org) was developed in French and later validated in participants with atypical syndromes. We report the validation of the English version.

**Methods:**

This multicentre prospective validation study was conducted in 10 centers across Canada and the United States on 260 English-speaking participants aged over 50. We translated and modified the original French DCQ to add targeted stimuli to the Visusopatial Index and social cognition vignettes to the Behavioral Index. A backward translation was performed and equivalence between languages was assessed by administering both tests to 30 bilingual participants.

**Results:**

Mean DCQ total score (out of 100) was 95.0 (SD = 3.6). Spearman’s correlation coefficient showed a strong and significant correlation (r = 0.49, *p* < .001) with the Montreal Cognitive Assessment. Test–retest reliability was good (Spearman’s coefficient = 0.72, *p* < .001) and interrater reliability, excellent (intraclass correlation = 0.97, *p* < .001). Normative data shown in percentiles were stratified by age and education for a population-based sample of 260 English-speaking controls aged between 50 and 87 years old.

**Conclusions:**

Similar to the French version, the English DCQ proved to be a valid cognitive screening test. The original version was very sensitive to detect atypical dementias such as primary progressive aphasias, Alzheimer’s disease’ variants and syndromes along the frontotemporolobar degeneration spectrum. This 20-min test can be administered *à la carte* and offers an alternative to detailed comprehensive neuropsychological evaluations.

## INTRODUCTION

Early recognition of atypical dementias poses a challenge because these conditions primarily affect language, behavior or visuospatial skills as opposed to classic amnestic memory deficits. Current cognitive screening tests such as the Mini-Mental State Examination or the Montreal Cognitive Assessment (MoCA) were not developed for such purposes and often underestimate or overestimate the neurocognitive findings in addition to having several methodological shortcomings. In turn, this creates a delay in proper identification of atypical syndromes, initiation of treatment and adequate management.

New cognitive screening tools were developed along the 2011 criteria for atypical syndromes such as Alzheimer's Disease (AD) variants (amnestic, visual, language, and behavioral), frontotemporolobar degeneration (FTLD) syndromes (behavioral variant Frontotemporal Dementia or bvFTD, primary progressive aphasias, corticobasal syndrome and Progressive Supranuclear Palsy or PSP). Among them the French version of Dépistage Cognitif de Québec (DCQ; dcqtest.org) was specifically developed to better identify atypical dementing syndromes (Laforce et al., 2018). Validation was conducted on a population-based sample of 410 healthy participants. The test demonstrated excellent psychometric properties equivalent to those of other tests considered as gold standard in cognitive screening. We further studied the DCQ in patients with various types of typical and atypical dementia and showed better sensitivity and specificity for atypical dementia than the MoCA (Sellami et al., 2018). Indexes were strongly correlated with standard neuropsychological evaluation and the DCQ helped distinguish atypical from typical dementias.

We present herein a multicenter study on the validation of the English version of the DCQ.

## METHODS

### Design

**Fig. 1:**
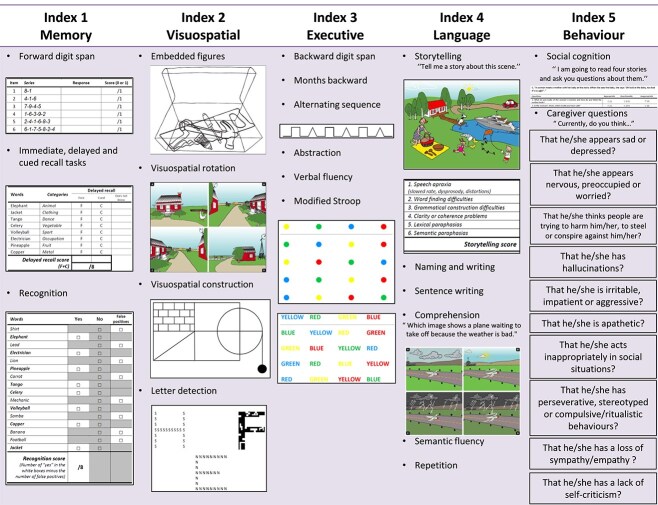
Illustrative summary of the subtests composing the five DCQ indexes.

Two independent linguists translated the original French DCQ to English (see Fig. 1). A backward translation was performed and equivalence between languages was assessed by administering both tests to 30 bilingual participants. Discrepancies between the two translations were assessed by a third translator.

We further added targeted stimuli to the Visusopatial Index (letter detection) and four social cognition vignettes based on the Faux Pas paradigm to the Behavioral Index of the DCQ. This was performed in an attempt to better capture visuospatial skills as well as neurobehavioral symptoms of atypical syndromes. Scoring ranges of the four cognitive indices can be found in Table 1.

**Table 1:** Percentile norms for the English DCQ: memory, visuospatial, executive, and language indexes

**Index**	**Age**	**Education**	***n***	**Percentiles**												
				1	2	5	10	15	25	50	75	85	90	95	98	99
Memory		≤12	28	13	13	19	21	22	23	24	24	24	24	24	24	24
(max = 24)	50–59	>12	60	22	22	23	23	24	24	24	24	24	24	24	24	24
	60–69	>12	91	21	21	22	23	23	24	24	24	24	24	24	24	24
	≥70	>12	81	18	18	22	23	23	23	24	24	24	24	24	24	24
Visuo-		≤12	28	7	7	8	10	10	11	13	14	14	14	14	14	14
spatial	50–59	>12	60	9	9	10	11	13	14	14	14	14	14	14	14	14
(max = 14)	60–69	>12	91	6	9	10	11	11	12	14	14	14	14	14	14	14
	≥70	>12	81	8	9	9	10	11	12	14	14	14	14	14	14	14
Executive		≤12	28	5	5	5	5	6	6	8	9	9	10	10	10	10
(max = 10)	50–59	>12	60	4	6	6	7	7	8	9	10	10	10	10	10	10
	60–69	>12	91	5	5	6	7	7	8	9	9	10	10	10	10	10
	≥70	>12	81	4	5	6	6	7	8	9	10	10	10	10	10	10
Language		≤12	28	19	19	21	23	23	24	26	27	27	27	27	27	27
(max = 28)	50–59	>12	60	22	22	25	26	26	27	27	28	28	28	28	28	28
	60–69	>12	91	23	24	25	25	26	26	27	28	28	28	28	28	28
	≥70	>12	81	23	24	24	25	26	26	27	28	28	28	28	28	28

*The median score for the Behavioural Index was 24 (max = 24) and therefore percentile ranks are not reported.

We then conducted a multicenter prospective effort in ten centers across Canada (Clinique Interdisciplinaire de Mémoire du CHU de Québec, Sunnybrook Research Institute at the University of Toronto, Clinic for Alzheimer’s Disease and Related Disorders at the University of British Colombia, McGill Research Centre for Studies in Aging, Hotchkiss Brain Institute at the University of Calgary, Sherbrooke Research Center on Aging, Horizon Health Network in St-John New Brunswick) and the United States (Alzheimer’s Disease Research Center at the University of California San Diego).

### Participants and recruitment

A sample of 260 English-speaking participants over 50-year-old was recruited among the general population. The local ethics committee approved the study protocol and all participants provided written informed consent. Participants were excluded if they reported a history of traumatic brain injury, delirium, brain surgery, neurological disease, encephalitis, meningitis, untreated metabolic condition, psychiatric illness, brain oncological therapy, alcohol or drug abuse, disabling visual or hearing impairments, and if they were receiving experimental therapy. They were also excluded if they were unable to undertake the test or if they were illiterate. They had no cognitive complaints and had preserved activities of daily living.

All participants completed the English DCQ and the MoCA on the same day. Thirteen DCQ-trained psychometricians across ten centers administered the DCQ and the MOCA. A sample of 15 participants was retested within 1–3 months of initial administration to examine test–retest reliability. And 25 questionnaires were picked at random among the 260 participants and scored again by two blinded and independent raters to assess interrater reliability. Twenty individuals completed a full neuropsychological evaluation and we compared their results to those of the DCQ Indexes.

## RESULTS

Basic descriptive analyses included means and standard deviations. Student T test was used to compare means. Reliability was tested for internal consistency using Cronbach’s alpha coefficient where a value >0.70 was considered appropriate. Test–retest reliability was assessed using Spearman’s correlation coefficient. An interrater reliability analysis using the intraclass correlation coefficient was performed to determine consistency among raters. Validity was established through correlations between DCQ total score (out of 100) and MoCA total score (out of 30) using Spearman’s correlation coefficient. Statistical analysis was performed using SPSS software (version 24.0) with the alpha level set at 0.05.

Mean age was 66.2 (SD = 8.1) while mean education was 16.7 (SD = 3.4). The mean DCQ total score out of 76 (i.e., without the Behavioral Index scored on 24) was 71.3 (SD = 3.7). The mean total score for the Behavioral Index in our sample of 260 normal participants was 23.4 (SD = 1.1) and the median was 24.

### Normative data

Normative data shown in percentiles were stratified by age and education for a population-based sample of 260 English-speaking controls aged between 50 and 87 years old (see Table 1). Education level was divided dichotomously into ≤12 and > 12. There were no differences between men and women on DCQ total score. To use this table, one should select the appropriate row corresponding to the patient’s age range, then select the patient’s education level, and finally find the patient’s raw score and refer to the corresponding percentile rank. The median is the 50th percentile.

### Validity and reliability

Validity of the DCQ was assessed by correlating performance on the DCQ total score to the MoCA total score. Spearman’s correlation coefficient showed a strong and significant correlation (r = 0.49, *p* < .001) with the Montreal Cognitive Assessment. Although based on a small sample of 15 participants, test–retest reliability was good (Spearman’s coefficient = 0.72, *p* < .001) and interrater reliability, excellent (intraclass correlation = 0.97, *p* < .001). Acceptability of the DCQ was good. The test was well tolerated by the participants, and the behavioral questionnaire was easily understood by the significant other.

## DISCUSSION

We previously developed a new cognitive screening test adapted to updated dementia criteria for AD (McKhann et al., 2011), PPA (Gorno-Tempini et al., 2011) and the FTLD spectrum (Rascovsky et al., 2011) that is valid and reliable (see dcqtest.org; Laforce et al., 2018). We further studied the DCQ in patients with various types of typical and atypical dementia and showed better sensitivity and specificity for atypical dementia than the MoCA (Sellami et al., 2018). Indexes were strongly correlated with standard neuropsychological evaluation and the DCQ helped distinguish atypical from typical dementias. We report the validation of the English version of the DCQ through a multicentre prospective validation in ten centers across Canada and the United States on 260 English-speaking participants aged over 50.

We translated and modified the original French DCQ to add targeted stimuli to the Visusopatial Index and social cognition vignettes to the Behavioral Index. A backward translation was performed and equivalence between languages was assessed by administering both tests to 30 bilingual participants.

We acknowledge that in this process, no precise adaptation was made to address cultural and environmental variations between our French-speaking and English-speaking populations. We aimed to provide clinicians with a more advanced instrument that allows in-depth testing of various cognitive domains *à la carte*. The five DCQ indexes were specifically designed to provide advanced information on specific cognitive domains. For example, the Language Index assesses semantic knowledge through confrontation naming and comprehension tasks. It also allows the identification of surface dyslexia/surface dysgraphia through writing and spelling of irregular words. Such features can be found in the semantic variant PPA. Other salient language deficits, such as poor word retrieval and impairment in repetition of long sentences seen in the logopenic variant of PPA, agrammatism in spoken and written production seen in nonfluent variant PPA or rating of apraxia of speech in spontaneous speech (Gorno-Tempini et al., 2011) are also tested within this index. The Visuospatial Index includes subtests that explore visual orientation and space perception without interference of executive and visuoconstructive skills on pure visuospatial functions. Following this rationale, we added a letter detection task to the original DCQ’s Visuospatial Index to allow better screening of the deficits associated with the visual variant of AD (also known as Posterior Cortical Atrophy) (Crutch et al., 2017) or Lewy Body dementia (McKeith et al., 2017). The Memory index includes immediate, delayed and cued recall tasks using the Dubois paradigm (Dubois et al., 2002). This method is known to better discriminate memory consolidation impairments seen in amnestic AD from other memory disorders. Finally, the Behavioral Index now includes social cognition vignettes which may help to capture subtle behavioral changes.

We warn clinicians that both the French DCQ and English DCQ are different tests with different stimuli, different scoring, normative percentile values, and schooling divisions. Both tests can be downloaded for free at dcqtest.org; the English version is located in the top right corner on our web site.

In conclusion, this study provided normative data for the English DCQ. This cognitive screening test is adapted to the new dementia criteria for AD variants, PPAs, bvFTD and the FTLD spectrum, and Lewy body dementia. The DCQ items were designed specifically to detect cognitive patterns associated with atypical dementias. It demonstrated excellent psychometric properties. Similar to our validation study of the French DCQ on clinical populations (Sellami et al., 2018), further studies are already underway to validate the English DCQ on samples of patients with atypical dementias. Finally a Spanish version of the DCQ is already validated and submitted for publication (Fernández-Romero et al, in press).
